# Dissecting the Dual Role of the Glial Scar and Scar-Forming Astrocytes in Spinal Cord Injury

**DOI:** 10.3389/fncel.2020.00078

**Published:** 2020-04-03

**Authors:** Tuo Yang, YuJuan Dai, Gang Chen, ShuSen Cui

**Affiliations:** ^1^Department of Hand Surgery, China-Japan Union Hospital of Jilin University, Changchun, China; ^2^Medical School of Nantong University, Nantong, China; ^3^Department of Tissue and Embryology, Medical School of Nantong University, Co-innovation Center of Neuroregeneration, Nantong University, Nantong, China; ^4^Department of Anesthesiology, Affiliated Hospital of Nantong University, Nantong, China

**Keywords:** spinal cord injury, glial scar, astrocyte, scar-forming astrocyte, regeneration

## Abstract

Recovery from spinal cord injury (SCI) remains an unsolved problem. As a major component of the SCI lesion, the glial scar is primarily composed of scar-forming astrocytes and plays a crucial role in spinal cord regeneration. In recent years, it has become increasingly accepted that the glial scar plays a dual role in SCI recovery. However, the underlying mechanisms of this dual role are complex and need further clarification. This dual role also makes it difficult to manipulate the glial scar for therapeutic purposes. Here, we briefly discuss glial scar formation and some representative components associated with scar-forming astrocytes. Then, we analyze the dual role of the glial scar in a dynamic perspective with special attention to scar-forming astrocytes to explore the underlying mechanisms of this dual role. Finally, taking the dual role of the glial scar into account, we provide several pieces of advice on novel therapeutic strategies targeting the glial scar and scar-forming astrocytes.

## Introduction

The past decades have witnessed a rapid increase in studies on the pathology and molecular mechanisms of and therapeutic strategies for spinal cord injury (SCI). The clinical management of SCI, including surgical interventions, supportive therapies and rehabilitation methods, markedly improves functional recovery and reduces disability (Fehlings et al., 2017). Due to the high complexity and limited recoverability of the human central nervous system (CNS), however, more than 27 million SCI patients worldwide remain disabled and experience decades of life with permanent disabilities and suffering (James et al., 2019). The reasons why the outcomes of human SCI remain so poor are complex and varied. The glial scar, a dense limiting border around the SCI lesion core (the lesion core is also known as the fibrotic scar) formed predominately by scar-forming astrocytes after SCI, has long been considered one of the primary causes of the failure of spinal cord regeneration.

Astrocytes, a major CNS component, are glial cells characterized by a star shape. Astrocytes provide essential physiological insulation and support for neurons. Their pathological importance was underestimated at first. In recent decades, however, astrocytes have been found to not only extensively participate in biological activities in the CNS, including the regulation of the blood-brain barrier, synaptic function and glutamate uptake (Füchtbauer et al., 2011; Haj-Yasein et al., 2011; Murai and Pasquale, 2011; Min and Nevian, 2012; Murphy-Royal et al., 2015), but also play crucial roles in pathological processes, including neurodegenerative diseases, stroke and CNS injuries (Bradford et al., 2009; Kuchibhotla et al., 2009; Wanner et al., 2013; Khakh and Sofroniew, 2015; Malik et al., 2020). After SCI, astrocytes are activated, and some of them rapidly proliferate to form an astrocytic scar border, traditionally referred to as the glial scar. The glial scar and scar-forming astrocytes play key roles in the recovery of SCI. A thorough analysis of the roles of the glial scar in SCI may help provide new views of SCI and identify novel therapeutic strategies. For decades, the glial scar was thought to be a primary inhibitor of SCI recovery (Sugar and Gerard, 1940; Clemente and Windle, 1954). However, this concept has been continually challenged in recent years since several studies have shown that the radical ablation of the glial scar failed to promote SCI recovery (Anderson et al., 2016; Gu et al., 2019). It is increasingly accepted that the glial scar plays a dual role in the pathological process of SCI, both protective and inhibitory. However, the complexity of this dual role has been underestimated. Many components of the glial scar, such as astrocytes, microglia, and oligodendrocyte precursor cells, are highly plastic. They not only directly contribute to the dual role of the glial scar but also interact with other components, increasing its complexity. Furthermore, this dual role is dynamic and changes as SCI progresses. This diverse and dynamic nature makes it difficult to target the glial scar for therapeutic purposes, resulting in a need to understand and take into account the dual role of the glial scar.

In the context of SCI, we dissect the dual role of the glial scar over time to explore the underlying mechanisms of its diverse and dynamic nature, with a focus on the disparity, variation, and interactions of scar-forming astrocytes. Taking the dual role of the glial scar into account, we analyze some potential interventions targeting the glial scar (especially scar-forming astrocytes) and offer several pieces of advice for novel therapeutic strategies.

## Timeline of Glial Scar Formation and The Transformation of Scar-Forming Astrocytes

### Astrocytic Activation Within Hours

Regardless of the cause of SCI, injury directly damages neural tissue and induces tissue ischemia, which is caused by vascular trauma and tissue swelling (Tator and Koyanagi, 1997; Mautes et al., 2000; Norenberg et al., 2004). These damaged cells release ATP, which acts on a variety of purinergic receptors expressed by astrocytes, microglia, oligodendrocytes and oligodendrocyte precursor cells (OPCs) and triggers the activated states of these cells (Franke et al., 2001; James and Butt, 2002). Additionally, astrocyte activation can also be triggered and amplified by multiple inflammatory factors, including TNF, IFN-γ, IL-6, and IL-1β (Liddelow et al., 2017). Astrocyte activation may occur immediately after SCI, since many studies show that some indicators of astrocyte activation [glial fibrillary acidic protein (GFAP), GFAP mRNA, pSTAT3, NF-κB and chondroitin sulfate proteoglycans (CSPGs)] significantly increase within a few hours of CNS insults, in both brain injuries and SCI (Brambilla et al., 2005, 2009; O’Callaghan et al., 2014; Takano et al., 2014). The early astrocyte activation in SCI is even stronger than that in brain injuries (Schnell et al., 1999). Morphologically, most reactive astrocytes are essentially similar to normal astrocytes in the uninjured spinal cord, but reactive astrocytes differ from normal ones in cellular hypertrophy and overexpression of GFAP (Wanner et al., 2013).

### Glial Scar Formation Within Days

Beginning at 1–2 days after injury, some reactive astrocytes rapidly proliferate and densely populate the area around the lesion core within a 7–10-day period of glial scar formation (Wanner et al., 2013). The proliferation of these astrocytes is the fastest 3–5 days after injury, slows 7 days after injury and is nearly stopped 14 days after injury. During this process, these astrocytes begin to extend elongated processes perpendicularly towards the lesion core. They gradually lose their domains and become dense to form a narrow (only several cell layers) limiting border (the glial scar) surrounding the lesion core (Herrmann et al., 2008; Wanner et al., 2013; Hara et al., 2017). Thus, these astrocytes transform into scar-forming astrocytes, which are quite different from reactive astrocytes in adjacent neural tissue (Khakh and Sofroniew, 2015). It is worth mentioning that most scar-forming astrocytes are newly proliferated (Zai and Wrathall, 2005). A recent quantitative analysis confirmed this view and showed that the number of astrocytic cell bodies in the glial scar is nearly twice that in the uninjured spinal cord. Additionally, this study also found a diminishing gradient of the proliferation and density of scar-forming astrocytes as the distance to the lesion core increases (Wanner et al., 2013). This topographical heterogeneity of scar-forming astrocytes and directionality of elongated processes indicate that reactive astrocytes may receive signals from the lesion core and phenotypically change into scar-forming astrocytes in response.

### Glial Scar Maturation Within Weeks

The rapid proliferation of reactive astrocytes gradually stops approximately 2 weeks after injury. From 1 to 2 weeks to several weeks after injury, the glial scar becomes completely mature (Ren et al., 2017). Scar-forming astrocytes complete their phenotypic change and no longer orient their processes perpendicularly towards the lesion core but instead become more parallel and overlap with each other. Thus, they completely lose their domains and form a compact mature glial scar (Wanner et al., 2013). After this point, the SCI lesion begins to stabilize, and a long chronic phase of regeneration begins. It is worth mentioning that the STAT3 pathway, a master regulator of astrocyte activation and glial scar formation, also controls glial scar maturation (Ceyzériat et al., 2016; Escartin et al., 2019). In STAT3-KO mice, the elongated processes of scar-forming astrocytes remain perpendicular to the lesion core and do not become parallel to form a dense astrocytic scar border after SCI (Wanner et al., 2013).

## Other Representative Components Associated with Scar-Forming Astrocytes

In addition to astrocytes, other components of the glial scar also play important roles in its formation. These components interact extensively with astrocytes. Some of them directly act on astrocytes, some are regulated by astrocytes and some are released by astrocytes. Although they are in the minority compared with scar-forming astrocytes, these components influence the activity and function of scar-forming astrocytes as well as other cell types and molecules in the glial scar. Following is a discussion of several representative components associated with scar-forming astrocytes and the interactions between them.

### Microglia

As the most sensitive responders to CNS injuries, microglia have a relatively low activation threshold and are the first reactive cell type in the cascade reactions following injuries (David and Kroner, 2011; Bellver-Landete et al., 2019). Microglia reach the maximum level of proliferation in the first week after SCI and are more beneficial during this period (Bellver-Landete et al., 2019). During the acute and subacute phase, microglia participate in the inflammatory response, phagocytosis for debris clearance (Greenhalgh and David, 2014), and in interactions with monocyte-derived macrophages to regulate inflammation (Greenhalgh et al., 2018). Glial scar formation highly depends on the interactions between reactive astrocytes and reactive microglia (Shinozaki et al., 2017). When activated, microglia release cytokines that trigger and maintain the activation of astrocytes. Microglia induce glial scar formation by releasing IGF-1, which triggers astrocytic activation and proliferation (Bellver-Landete et al., 2019). The *in vivo* inhibition of IGF-1 results in attenuated astrocytic activation and proliferation. The depletion of microglia also leads to attenuated glial scar formation and worse functional recovery, suggesting a crucial role for microglia in glial scar formation (Bellver-Landete et al., 2019). Another study demonstrates that microglia may downregulate the astrocytic P2Y1 receptor to improve glial scar formation after CNS injury (Shinozaki et al., 2017). There have also been reports indicating that microglia are responsible for inducing a neurotoxic phenotype of astrocytes (Liddelow et al., 2017). In the chronic phase, microglia remain remarkably activated across the entire spinal axis up to 180 days after SCI and participate in the chronic neuropathic pain (Chen et al., 2018).

### Macrophages

The astrocyte-macrophage axis in SCI is complex, dynamic and crucial for recovery. In the acute phase, reactive astrocytes participate in the early inflammatory response including recruiting monocytes from peripheral blood to the lesion site by releasing multiple cytokines and chemokines (e.g., CCL2, CCL5, CXCL8; Mildner et al., 2009; Shechter et al., 2013; Zhou et al., 2018). These monocytes will further differentiate into macrophages including two main subtypes, the pro-inflammatory subtype M1 and the anti-inflammatory subtype M2. This recruitment of macrophages lays a crucial foundation for recovery from SCI since these macrophages not only clear toxic debris from injured tissues through phagocytosis but also remove dying neutrophils through efferocytosis (Hawthorne and Popovich, 2011). Reactive astrocytes induce chemotaxis and polarization of macrophages to manipulate their fates (Li et al., 2020). In the subacute phase, macrophages amplify astrocytic activity and promote glial scar formation. *In vitro*, M1 macrophages are essential inducers of astrocytic activation (Haan et al., 2015). On the other hand, M2 macrophages secrete TGF-β to induce glial scar formation *in vitro* (Song et al., 2019). M2 macrophages may also enhance the polarization of reactive astrocytes after SCI (Sonn et al., 2019). Inhibition of the centripetal migration of macrophages impairs the migration of astrocytes and glial scar formation, resulting in exacerbated neuronal loss and decreased functional recovery (Kobayakawa et al., 2019). However, excessive macrophage activity also contributes to damaging cascade reactions, secondary damage and axonal dieback (Evans et al., 2014). M1 macrophages induce necroptosis of reactive astrocytes, which may be an important mechanism of secondary damage of SCI (Fan et al., 2016). Meanwhile, reactive astrocytes may act as negative feedback regulators that in turn inhibit macrophages’ activity. *In vitro*, M2 macrophage-stimulated astrocytes can inhibit the proliferation of both M1 and M2 macrophages and decrease the production of proinflammatory factors (Haan et al., 2015).

All of these results indicate that macrophages have a critical influence on glial scar formation and scar-forming astrocytes. Astrocytes also influence macrophages in turn, but the exact roles of M1 and M2 macrophages require more investigation. It is worth mentioning that most studies on macrophages in SCI focus on their roles in the acute and subacute phase, but they may still be active (Beck et al., 2010) and influence axonal regeneration in the chronic phase (Zukor et al., 2013).

### Chondroitin Sulfate Proteoglycans

After SCI, extracellular matrix (ECM) molecules undergo differential regulation, as some, like hyaluronan are degraded, some, like CSPGs, are upregulated and some, like tenascin-C, are newly expressed (Haggerty et al., 2017). These changes lead to the emergence and enrichment of some key inhibitory molecules and eventually make the ECM an inhibitory environment for regeneration after injury. Among these key inhibitory molecules, CSPGs are the most prominent. CSPGs, a proteoglycan family characterized by chondroitin sulfate side chains, are thought to be released predominantly from neurons and scar-forming astrocytes (Quraishe et al., 2018). It has long been thought that CSPGs are crucial inhibitory factors of CNS axonal regeneration, making them a primary cause of the inhibitory roles of the glial scar (McKeon et al., 1991). CSPGs mainly act on oligodendrocytes and neurons to exert their inhibitory function. Recent studies have proven that CSPGs inhibit axonal regeneration through excessive adhesion but not repulsion (McTigue et al., 2006; Filous et al., 2014; Silver and Silver, 2014; Lang et al., 2015). Mediated by several receptors, including phosphatase leukocyte common antigen-related (LAR), protein tyrosine phosphatase σ (PTPσ) and nogo receptors 1, 2, and 3 (NgR), CSPGs adhere to and immobilize regenerating axons to function as inhibitory factors (Sapieha et al., 2005; Schwab and Strittmatter, 2014; Lang et al., 2015; Dyck et al., 2018, 2019). Many studies have focused on the development of therapeutic strategies against CSPGs, including interventions targeting their chondroitin sulfate side chains or downstream receptors. These studies showed satisfactory results, making CSPGs a promising therapeutic target for SCI (further discussion is in “Strategies Targeting CSPGs” section). However, whether scar-forming astrocytes are the primary producers of CSPGs remains controversial (see “Scar-Forming Astrocytes Are Major, but Not Predominant, Producers of CSPGs” section).

### NG2-OPCs

NG2-OPCs, another cellular component of the glial scar, refer to OPCs that express neuron-glial antigen 2 (NG2; mainly on their cell membrane). OPCs rapidly react after SCI and exhibit cell process retraction, cell hypertrophy and increased expression of NG2 (Levine, 2016). As a member of the CSPG family, NG2 is a powerful axonal regeneration inhibitor (Ughrin et al., 2003), which makes NG2-OPC another major inhibitory CSPG producer besides scar-forming astrocytes in the glial scar (Dou and Levine, 1994). The inhibition of NG2 by an NG2 neutralizing antibody after SCI can improve spinal cord recovery (Tan et al., 2006; Petrosyan et al., 2013). Despite evidence indicating the inhibitory roles of the NG2 CSPG, NG2-OPCs themselves may not be inhibitory since several studies have indicated that NG2-OPCs are closely related to axonal regeneration (Yang et al., 2006; Filous et al., 2014; Hackett and Lee, 2016). NG2-OPCs may also play fundamental roles in glial scar formation, since the glial scar exhibits discontinuous morphology and decreased GFAP density after inhibition of NG2-OPCs (Rodriguez et al., 2014; Hesp et al., 2018). The inhibition of NG2-OPCs in these studies moderately reduces the density of the glial scar, but not as radically as in other studies of glial scar ablation (Anderson et al., 2016; Gu et al., 2019). Interestingly, this relatively moderate glial scar disruption induced by the NG2-OPC inhibition results in increased axon regeneration, which is quite different from the radical ablation (Rodriguez et al., 2014; Anderson et al., 2016; Hesp et al., 2018; Gu et al., 2019). When the inhibition of NG2+ cells was discontinued, the glial scar became compact again (Hesp et al., 2018). These studies on NG2-OPCs may provide us with clues to new interventions targeting the glial scar which moderately reduces its density without interrupting its integrity. It is worth mentioning that it is not known whether the axon regeneration results from NG2 inhibition itself or follows glial scar disruption. Also, NG2-OPCs can differentiate into astrocytes in injured CNS including in SCI (Raff et al., 1983; Sellers et al., 2009; Hackett et al., 2016). Interestingly, this differentiation of NG2-OPCs into astrocytes is mainly induced by the expression of bone morphogenetic proteins (BMPs) by reactive astrocytes, contributing to glial scar formation (Wang et al., 2011). Taken together, these studies on NG2-OPCs deepen our understanding of the complexity of the glial scar and provide us with new ideas to modulate the glial scar by manipulating the fate of NG2-OPCs.

## The Dual Role of The Glial Scar in Sci Recovery

**Figure 1:**
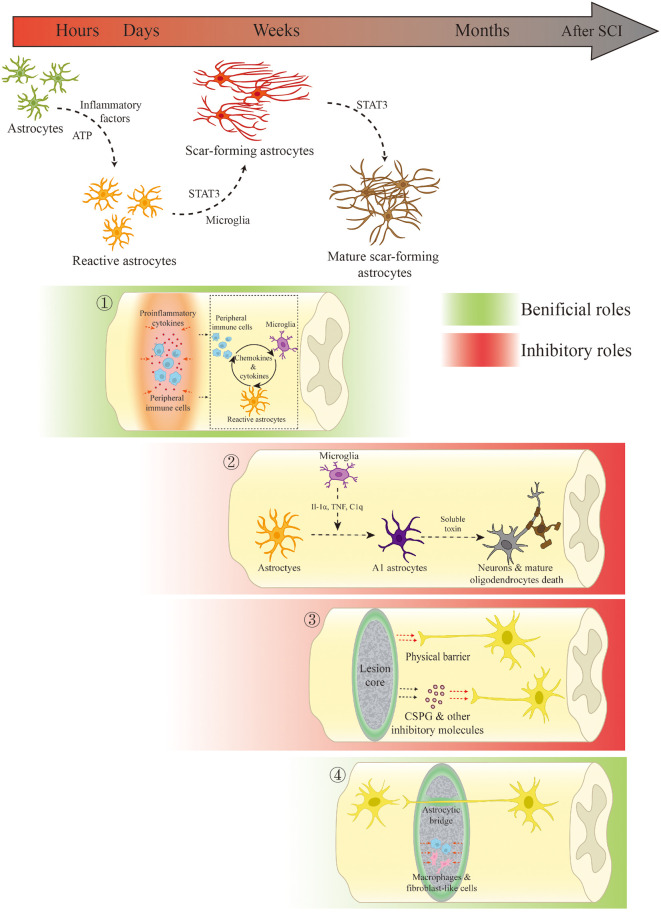
Scar-forming astrocyte transformation and the dual role of the glial scar and scar-forming astrocytes in spinal cord injury (SCI). ① In the acute and subacute phases of SCI, the glial scar and scar-forming astrocytes not only restrict the spread of inflammation but also regulate inflammation through interactions with innate and peripheral immune cells. ② Microglia-mediated A1 phenotypic changes result in neuronal and mature oligodendrocytic death through the release of soluble toxins. ③ The glial scar inhibits spinal cord regeneration through physical obstruction and the release of inhibitory molecules. ④ In the chronic phase, the glial scar persistently limits the fibrotic tissue and macrophages. Scar-forming astrocytes may serve as bridges for axonal growth under certain conditions.

Many studies have attempted to demonstrate the roles of the glial scar in SCI in recent years (Table 1). While the exact roles of the glial scar in SCI remain unclear, it has become widely accepted that the glial scar plays a dual role in SCI, both protective and inhibitory. According to the timeline of SCI development and glial scar formation, we summarize the dual role of the glial scar (Figure 1).

**Table 1:** Recent studies demonstrating the roles of the glial scar in SCI.

Role of the glial scar in SCI	SCI model	Intervention	Results	Reference
Aids regeneration	T10 forceps crush injury (mice)	Inhibition of the glial scar by STAT3 knockout, TK/GCV, and diphtheria toxin-mediated astrocyte ablation	Failed to result in spontaneous axonal regrowth	Anderson et al. (2016)
	T8 forceps crush injury (mice)	Selective ablation of the glial scar by the HSV-TK/GCV system	Failed to improve spontaneous functional recovery	Gu et al. (2019)
	C5 contusion (mice)	Glial scar disruption by NG2 ablation	Impaired forelimb locomotion	Hesp et al. (2018)
Restricts inflammatory and fibrotic cells	L1/L2 forceps crush injury (mice)	Selective deletion of STAT3	Increased the spread of inflammatory and fibrotic cells; increased neuronal loss	Wanner et al. (2013)
	T10 aneurysm clip crush injury (mice)	Conditional ablation of astrocytic BMPR1a	Reduced astrocytic hypertrophy, increased inflammatory infiltration and reduced axon density	Sahni et al. (2010)
	L1/L2 longitudinal stab injury and moderate crush injury	Ablation of reactive astrocytes by the HSV-TK/GCV system	Failure of blood-brain barrier repair, leukocyte infiltration, local tissue disruption, severe demyelination, neuronal and oligodendrocyte death and pronounced motor deficits	Faulkner et al. (2004)
Provides permissive bridges for axonal regeneration	T8 forceps crush injury (mice)	shRNA-mediated PTEN suppression	Most axons regrew along the astrocytic bridge	Zukor et al. (2013)
Induces A1 astrocytes, which kill neurons and mature oligodendrocytes	T10 weight-drop impact injury (rats)	Notch signaling pathway blockage	Suppresses A1 astrocyte transition	Qian et al. (2019)
	T10 impactor contusion injury (rats)	Intravenous injection of mesenchymal stem cells or their exosomes	Decreased lesion area and improved motor function	Wang L. et al. (2018)
Inhibits axonal regeneration	T10 impactor contusion injury (mice)	Reduction in glial scar formation through the pharmacological blockade of astrocytic type I collagen interaction	Improved axonal regeneration and functional recovery	Hara et al. (2017)
Produces CSPGs to inhibit spinal cord regeneration	T10 impactor contusion injury (mice)	Chondroitin sulphate N-acetylgalactosaminyl-transferase-1 gene knockout	Improved recovery compared to that of chondroitinase ABC-treated mice and wild-type mice	Takeuchi et al. (2013)
	T8 impactor contusion injury (rats)	CSPG receptor blockade by a CSPG receptor PTPσ mimetic peptide	Facilitated functional recovery	Lang et al. (2015)
	C7 hemisection (rhesus monkeys)	Intraparenchymal injections of chondroitinase	Improved hand function	Rosenzweig et al. (2019)
	C2 hemisection (rats)	A combined chondroitinase ABC and intermittent hypoxia conditioning treatment	Led to a rapid and robust respiratory and motor recovery	Warren et al. (2018)
	T8 hemisection (rats)	Decreasing CSPGs and fibrotic scarring by microtube stabilization	Promoted axonal regeneration	Hellal et al. (2011)

### Inhibitory Roles

#### The Glial Scar as a Physical Barrier to Inhibit Axonal Regrowth

For decades, the glial scar had been considered a critical inhibitor of CNS regeneration. Some early observations showed that regenerating axons fail to grow past the glial scar, leading to the hypothesis that the glial scar forms a physical barrier that obstructs axonal regeneration (Sugar and Gerard, 1940; Brown and McCouch, 1947). Several studies provided more evidence for this hypothesis, thus making it prevalent (Clemente and Windle, 1954). However, it has also been challenged for decades due to the inability to replicate the beneficial effects of glial scar attenuators (Arteta, 1956; Matthews et al., 1979) and more recent observations that the ablation of the glial scar fails to facilitate spinal cord regeneration (Anderson et al., 2016). However, although many researchers have already focused on other inhibitory mechanisms, the notion of the glial scar as a physical barrier has persisted until today and remains controversial. The confirmation or rejection of this notion may be challenging but is necessary.

#### Scar-Forming Astrocytes Are Major, but Not Predominant, Producers of CSPGs

Another reason why the glial scar was previously considered a crucial inhibitor is that the glial scar participates in building an inhibitory ECM for axonal regeneration by permanently producing several inhibitory molecules, including CSPGs. Astrocytes express increased levels of CSPGs after SCI and were previously thought to be the predominant producers of CSPGs (McKeon et al., 1999; Jones et al., 2003). Selectively isolated scar-forming astrocytes also exhibit high levels of expression of CSPGs, which provides additional evidence for this line of thought (Hara et al., 2017). However, scar-forming astrocytes may not be the predominant producers of CSPGs since the radical ablation of scar-forming astrocytes fails to reduce the total CSPG level in SCI lesions; the lesion area fills with GFAP-negative but CSPG-positive cells after astrocyte ablation (Anderson et al., 2016). Other cell types, such as NG2-OPCs, macrophages, and oligodendrocytes also contribute to CSPG enrichment (Uhlin-Hansen et al., 1993; Asher et al., 2002; Tan et al., 2005). Some studies ablating particular CSPG-producing cell types (including scar-forming astrocytes and NG2-OPCs) failed to improve axonal regeneration (Kucharova et al., 2011; Filous et al., 2014; Anderson et al., 2016). These findings may be explained by the observations that: (1) there is no dominant CSPG producer, but most of the cellular components of the glial scar contribute to CSPG enrichment; and (2) scar-forming astrocytes and other glial cells are highly plastic and may be complementary in expressing CSPGs. Thus, directly targeting CSPGs or targeting the downstream receptors of CSPGs would be a better choice than the ablation of particular CSPG-producing cell types for therapeutic interventions to regulate CSPGs.

#### The Neurotoxic Phenotype of Reactive Astrocytes

Based on the plasticity and heterogeneity of astrocytes that accumulating evidence has suggested (Adams and Gallo, 2018), it is reasonable to infer that there may be particular astrocytic phenotypes playing a neurotoxic or neuroprotective role which contribute to the dual role of the glial scar. A neurotoxic subgroup of reactive astrocytes, which was termed the A1 phenotype, has been found in brain injuries and neurodegenerative disorders recently (Liddelow et al., 2017). The study by Liddelow et al. (2017) indicates that reactive microglia releases cytokines such as Il-1α, TNF and C1q to induce A1 astrocytes. The powerful neurotoxicity of A1 astrocytes can kill oligodendrocytes and neurons *in vitro*. The *in vivo* blockade of A1 astrocyte formation through a total KO of C1qa/TNF/IL1a prevents the death of CNS neurons. Similar to the results in brain injury models, a recent study demonstrated the existence and neurotoxic function of A1 astrocytes in an SCI model (Qian et al., 2019). In this study, the immunofluorescence of C3 (a marker of A1 astrocytes) increases as early as 3 days after SCI. At 28 days after SCI, it shows a large area of increased immunofluorescence of C3 around the lesion core, which is consistent with the distribution and topographical heterogeneity of scar-forming astrocytes. This finding indicates that A1 astrocytes may comprise part of the scar-forming astrocytes in SCI. Like the transition of scar-forming astrocytes, reactive astrocytes may receive inflammatory factors from the lesion site to transform into a neurotoxic phenotype in the context of SCI, which is consistent with the findings of Liddelow et al. (2017). In another study of A1 astrocytes in SCI, mesenchymal stem cells (MSC) and MSC-exosomes were used to reduce A1 astrocytes through downregulating inflammatory factors (Wang L. et al., 2018). Wang L. et al. (2018) also found a better functional recovery by reduction of A1 astrocytes. However, it is worth mentioning that in these studies, the single marker C3 which the authors used may not be sufficient to qualify astrocytes as “A1” and neurotoxic. The direct relationship between the reduction of A1 astrocytes and functional recovery also needs more proof. Qian et al. (2019) changed the astrocytic state through interventions on the Notch-STAT3 axis, which was quite different from the study of Liddelow et al. (2017). Regardless, these findings suggest that: (1) the existence of a neurotoxic/neuroprotective phenotype of astrocytes in the context of SCI is reasonable; (2) the A1 astrocytes may be a part of the scar-forming astrocytes and contribute to the inhibitory roles of the glial scar; (3) the A1 astrocytes are closely related to inflammation and are induced as early as the acute phase of SCI; and (4) although the existence, biomarkers, and behavior of A1 astrocytes remain controversial, it may provide a new way to understand the neuronal loss and axonal dieback in SCI. The identification and remodeling of A1 astrocytes in SCI are worth further investigation.

### Beneficial Roles

#### The Glial Scar and Reactive Astrocytes Contribute to the Balance of Inflammatory Activities in the Acute and Subacute Stages of SCI

Debris clearance mediated by inflammatory cells is essential for recovery. However, the uncontrolled spread of cytotoxic inflammation can be devastating for spared neural tissues. Although the roles of the glial scar are still unclear, there is a consensus that the glial scar restricts the spread of inflammation to protect spared neural tissues (Sofroniew, 2015). Previous studies have demonstrated a key role of astrocytic migration in the healing process after SCI. Limiting astrocytic migration by conditional ablation of STAT3 leads to an enlarged lesion area, increased inflammatory infiltration and impaired recovery of motor function, whereas facilitating astrocytic migration by conditional ablation of SOCS3 results in enhanced contraction of the lesion area and improved functional recovery (Okada et al., 2006). Ablation of the glial scar in the acute or subacute phase also leads to an increased lesion range and worse recovery (Anderson et al., 2016). These studies show a critical role of the glial scar in limiting inflammation and indicate that inhibition of glial scar formation in the acute or subacute phase does not help or even worsens recovery. However, the glial scar is not only anti-inflammatory. After SCI, reactive astrocytes, microglia and peripheral immune cells release proinflammatory cytokines and interact with each other, which contributes to the inflammation in the acute and subacute stage (Escartin et al., 2019). Reactive astrocytes also have negative feedback regulation for inflammation after SCI (Haan et al., 2015; Zhang et al., 2019). In summary, the glial scar and reactive astrocytes are not mere limiters of the inflammation but rather balancers which are not only limiting but also contributing to the inflammation.

#### The Glial Scar Serves as a Restrictive Border to Limit Fibrotic Tissue and Macrophages After the Acute Stage of SCI

Many scientists have proposed that the glial scar may become more harmful than beneficial in the chronic phase of SCI. The early ablation of the glial scar fails to improve recovery, but are delayed interventions feasible? It is unlikely, based on the findings of Anderson and colleagues, who found that the ablation of the chronic glial scar mediated by diphtheria toxin 5 weeks after injury fails to help axons regenerate (Anderson et al., 2016). This result, however, may not lead to the conclusion that the chronic glial scar directly aids SCI recovery. The ablation of the chronic glial scar and adjacent reactive astrocytes causes enlarged lesions and tissue degeneration (Anderson et al., 2016). This finding may tell us that the fibrotic tissues and macrophages in the lesion core stay active and are influential not only in the acute and subacute phase but also in the chronic phase; they may also be inhibitory factors for axonal regeneration, since regenerating CST axons avoid clusters of these cells (Zukor et al., 2013) and decreased chronic fibrotic response improves axonal regeneration and functional recovery (Cooper et al., 2018). Therefore, after the radical ablation of the chronic glial scar, fibrotic tissues and macrophages in the lesion core may impair axonal regeneration and enlarge the lesion without limitation by the glial scar and adjacent reactive astrocytes. This inference is consistent with the results of Anderson et al. (2016). Interestingly, many studies indicate that the removal of chronic scar tissue improves axonal regeneration (Li et al., 2018; Patil et al., 2018; Wang N. et al., 2018). However, the scar tissue removed in these studies includes both the glial scar and the fibrotic scar. This scar removal eliminates the harmful effects from the fibrotic scar, and no more limitation is needed for them, promoting axonal regeneration.

In summary, fibrotic tissues and macrophages in the lesion core are still active and detrimental in the chronic phase of SCI. The glial scar and adjacent reactive astrocytes may contribute to limit the detrimental effects of fibrotic tissue and macrophages. The radical ablation of the chronic glial scar may fail to help since the benefits provided by the ablation may not be sufficient to overcome the inhibitory factors of uncontrolled fibrotic tissues and macrophages. It is worth mentioning that this inference may need more direct evidence and the roles of the chronic glial scar deserve more attention.

#### Scar-Forming Astrocytes Exhibit Environment-Dependent Plasticity and Could Serve as Bridges for Axonal Regrowth Under Certain Conditions

Previous studies have found the following: in physiological development, axons grow along immature astroglia (Mason et al., 1988; Raper and Mason, 2010); the glial scar is mostly formed by newly proliferated astrocytes with immature astroglia properties (Wanner et al., 2013); in lower vertebrates, axons regenerate along newly proliferated immature astroglia (Mokalled et al., 2016); immature astroglia grafts support axonal regrowth across the lesion core (Davies et al., 2006; Shih et al., 2014). According to these findings, it is reasonable to infer that scar-forming astrocytes could be permissive for axonal growth or even serve as bridges.

The bridge formed by astrocytes is more like a short “drawbridge (Silver, 2016).” Scar-forming astrocytes could lay down their “drawbridge” to let regenerating axons pass through only if the lesion core is artificially made to be thin enough (e.g., if it is induced by spinal cord crush with extremely fine forceps; Zukor et al., 2013). This study also shows that astrocytes that form astrocytic bridges are not derived from ependymal stem cells but are more likely derived from mature scar-forming astrocytes (Zukor et al., 2013). Thus, some scar-forming astrocytes are transformed into beneficial bridge-forming astrocytes through the artificial regulation of the environment, indicating that glial cells may have high environment-dependent plasticity. This environment-dependent plasticity was supported by another study in which the sequential phenotypic change in scar-forming astrocytes was demonstrated to be reversible, since reactive astrocytes could be reverted to naïve astrocytes when transplanted into the naïve spinal cord after SCI. On the other hand, naïve astrocytes formed astrocytic scars when transplanted into injured spinal cords (Hara et al., 2017). These findings provide us with a novel strategy for astrocytic reprogramming through environmental interventions instead of the direct manipulation of astrocytes.

## Potential Therapeutic Strategies Targeting Scar-Forming Astrocytes

Although the glial scar is more than merely scar-forming astrocytes, most current studies still focus on manipulating astrocytes. Here, we pose some promising strategies for discussion.

### Environmental Regulation of Astrocytic Fate

Accumulating findings have suggested the critical role of the ECM in SCI (Klapka and Müller, 2006; Schwab and Strittmatter, 2014; Yokota et al., 2017). ECM molecules not only directly participate in neuronal loss, inflammatory infiltration and axonal dieback/regrowth but also determine the outcomes of SCI by modulating scar-forming astrocytes and other cell types (Gaudet and Popovich, 2014; Didangelos et al., 2016; Bradbury and Burnside, 2019). As mentioned before, astrocytes exhibit high environment-dependent plasticity and are highly influenced by ECM molecules. For example, periostin, a type of ECM protein, is a key promotor of glial scar formation. Glial scar formation can be inhibited by the pharmacological blockade of periostin to improve functional recovery after SCI (Yokota et al., 2017). In another study, the pharmacological activation of Epac2 modulates the lesion environment and remodels reactive astrocytes to guide regenerating axons (Guijarro-Belmar et al., 2019). Also, changes in physical structure may influence astrocytic functions since scar-forming astrocytes could serve as bridges to allow regenerating axons to pass through when the injury width is sufficiently narrow (Zukor et al., 2013). Thus, remodeling astrocytes through environmental interventions is promising for therapeutic use.

### Direct Cell Reprogramming Strategies

*In vivo* direct cell reprogramming strategies once surprised us and have been widely investigated in CNS disorders (Guo et al., 2014; Trudler and Lipton, 2019). Since irreversible neuronal loss is another primary problem in SCI recovery, cell reprogramming may solve this problem by converting other cell types into neurons. Previous studies showed that *in vivo*, astrocytes can be reprogrammed into functional neurons after SCI through a single transcription factor (Su et al., 2014; Zarei-Kheirabadi et al., 2019). These reprogramming strategies exhibit limited efficiency in transforming astrocytes, thereby avoiding excessive attenuation of the glial scar. Therefore, with a more appropriate design, these astrocytic reprogramming strategies may be promising to not only supply functional neurons for the axonal relay but also modestly attenuate the density of the glial scar without interrupting its integrity. Unfortunately, few current studies have provided exciting behavioral results. One possible reason is that the time required for gene delivery and astrocytic reprogramming to provide functional neurons exceeds that required for the irreversible degeneration of target muscles. Rehabilitation strategies can provide use-dependent plasticity and reduce muscle degeneration, making them potential complementary therapies for astrocytic reprogramming strategies.

### Astrocytic Phenotype Remodeling Strategies

As mentioned before, the sequential phenotypic change in astrocytes is reversible (Hara et al., 2017). It is possible to attenuate neuronal loss and improve regeneration by remodeling astrocytic phenotypes. For instance, A1 astrocytes may widely participate in multiple pathological processes in CNS injuries and disorders (Liddelow et al., 2017). In models of neurodegenerative diseases, the neuronal loss and behavioral deficits were reduced by blocking microglia-mediated A1 astrocytic conversion (Yun et al., 2018). In the context of SCI, remodeling A1 astrocytes also led to better functional recovery (Wang L. et al., 2018). For future studies in this area, new markers for accurately identifying different phenotypes of astrocytes, detailed mechanisms of astrocytic phenotype conversion and the role of specific phenotypes in SCI may need further investigation.

### Strategies Targeting CSPGs

As mentioned before, CSPGs as the representative inhibitory molecules produced by scar-forming astrocytes are promising therapeutic targets for SCI. Current strategies mainly aim to target their chondroitin sulfate side chains or downstream receptors. Chondroitinase ABC, an enzyme that degrades the CSPG glycosaminoglycan chains, is a representative intervention targeting CSPGs. Chondroitinase ABC can attenuate the inhibitory activity of CSPGs and promote axonal regeneration (Bradbury et al., 2002; Pakulska et al., 2017). It can significantly propel functional recovery in many kinds of SCI models and can also be combined with other treatments (e.g., stem cell transplantation, peripheral nerve autografts, and conditioning treatment) to achieve better results (Alilain et al., 2011; DePaul et al., 2015; Suzuki et al., 2017; Warren et al., 2018). Other studies on chondroitinase ABC aim to innovate the delivery route to promote clinical applications (Burnside et al., 2018; Hu et al., 2018). The receptors of CSPGs are also potential targets for therapeutic strategies. Many studies show that manipulating these receptors can improve SCI recovery (Lang et al., 2015; Dyck et al., 2018). In general, therapeutic strategies targeting CSPGs are expected to provide a permissive microenvironment for spinal cord regeneration and become an important part of the comprehensive management of SCI.

### Comprehensive Management of SCI Through Combination Therapy Including Astrocytic Regulation

Many interventions are effective individually in the more regenerative rodent CNS. The regeneration of the human CNS, however, is more complex and challenging. More and more scholars propose that SCI may require comprehensive therapies and management to maximize recovery (Courtine and Sofroniew, 2019; Yu and Gu, 2019; Griffin and Bradke, 2020). Strategies targeting the glial scar may also require complementary strategies, because: (1) satisfactory spinal cord regeneration may need a combination of several key factors (Anderson et al., 2018) and (2) strategies targeting the glial scar may provide a permissive microenvironment for axonal regeneration, but several studies indicate that robust axonal regeneration may be insufficient to result in significant functional improvement because it also requires synaptic reorganization and use-dependent plasticity provided by rehabilitation or other engineering strategies (Zukor et al., 2013; Du et al., 2015; Anderson et al., 2018; Courtine and Sofroniew, 2019). As a relatively mature strategy targeting the glial scar, chondroitinase ABC has been used in many studies to investigate combination therapies (Griffin and Bradke, 2020). By removing the inhibitory microenvironment, chondroitinase ABC treatments amplify the effects of other treatments, leading to better recovery (Alilain et al., 2011; DePaul et al., 2015; Suzuki et al., 2017; Warren et al., 2018). Other strategies targeting the glial scar may also apply to combination therapy, and in general, combinations of glial scar manipulations may help to overcome some of the key challenges in enhancing SCI recovery.

## Summary and Recommendations for Future Study

Accumulating evidence has suggested that the glial scar may not be entirely harmful but may be a potential therapeutic target or may even provide new hope for spinal cord regeneration. Based on the aforementioned roles of the glial scar, a summary and recommendations for future study follow:

(1) Glial scar formation and scar-forming astrocytes may offer more benefits in the acute and subacute stages of SCI. Radical interventions on glial scar formation in the acute phase or even genetic ablation may not contribute to recovery (Anderson et al., 2016; Gu et al., 2019). Additionally, multiple factors (e.g., fractures, bleeding, tissue oedema, compression, risks of infection and inflammation) limit the clinical application of early interventions on the glial scar. Novel therapeutic strategies should be more cautious in manipulating astrocytes in the acute phase.
(2) Radical ablation of the glial scar may not help spinal cord regeneration since the glial scar is playing a dual role. The integrity of the glial scar provides a critical barrier to limit the spread of inflammation and the detrimental effects of fibrotic tissue and macrophages in the chronic phase. However, several studies resulted, intentionally or unintentionally, in a moderate reduction of the density of the glial scar. This reduction of the glial scar led to improved axonal regeneration and functional recovery (Rodriguez et al., 2014; Hesp et al., 2018; Zarei-Kheirabadi et al., 2019). These studies may provide us with clues as to how to manipulate the glial scar; that is, to reduce its density modestly without interrupting its integrity.
(3) Glial cells are highly plastic, which means they are highly influenced by environmental changes and exhibit different functions and roles. Functional changes in scar-forming astrocytes are not only causes but also consequences. Direct interventions on scar-forming astrocytes may fail to help regeneration without fundamentally regulating the cellular environment. It may be a better idea to modulate scar-forming astrocytes through manipulations on the microenvironment or downstream receptors.
(4) Recovery from SCI requires a combination of multiple essential factors, among which the least effective one may decide the outcome. *Anderson et al*. have once offered evidence for this theory by a study in which combined use of three facilitators significantly improved axon regeneration after SCI (Anderson et al., 2018). These facilitators are all required individually since the absence of any one element significantly reduces the effect and results in poor axon regeneration. As mentioned before, several studies have indicated that robust axonal regeneration may also be insufficient to result in significant functional improvement (Zukor et al., 2013; Du et al., 2015; Anderson et al., 2018; Courtine and Sofroniew, 2019). One possible reason is the absence of other essential factors for recovery, including synaptic reorganization and use-dependent plasticity. Based on this theory, it seems impractical to conquer multiple deficiencies in spinal cord regeneration through single interventions. The human CNS is more complex and less regenerative than the CNS of rodents, and single interventions may be effective in rodents but not in humans. To accelerate clinical translation, a more comprehensive management of SCI is worthy of consideration. Combined applications of complementary interventions that act on multiple targets at various time points deserve further investigation. The regulation of the glial scar may also serve as a critical part of the comprehensive management of SCI.

## Conclusions

The critical role of the glial scar in SCI has been gradually and widely accepted. The glial scar, however, is more complex than we initially thought. It contains multiple cellular components and a complex ECM. It plays multifaceted roles in SCI that are highly dynamic and change based on time, position, environment and therapeutic intervention. These properties of the glial scar and scar-forming astrocytes provide tremendous therapeutic potential. Since the glial scar plays a dual role, novel therapeutic strategies should attenuate its inhibitory roles while maintaining or amplifying its beneficial roles. To accelerate clinical translation, combination therapies involving glial scar interventions deserve consideration.

## Author Contributions

SC and GC contributed to the conception and design of the review. TY drafted the manuscript. TY and YD contributed to the revision of the manuscript.

## Conflict of Interest

The authors declare that the research was conducted in the absence of any commercial or financial relationships that could be construed as a potential conflict of interest.
