# Corrigendum: Dissecting the Dual Role of the Glial Scar and Scar-Forming Astrocytes in Spinal Cord Injury

**DOI:** 10.3389/fncel.2020.00270

**Published:** 2020-10-06

**Authors:** Tuo Yang, YuJuan Dai, Gang Chen, ShuSen Cui

**Affiliations:** ^1^Department of Hand Surgery, China-Japan Union Hospital of Jilin University, Changchun, China; ^2^Medical School of Nantong University, Nantong, China; ^3^Department of Tissue and Embryology, Medical School of Nantong University, Co-innovation Center of Neuroregeneration, Nantong University, Nantong, China; ^4^Department of Anesthesiology, Affiliated Hospital of Nantong University, Nantong, China

**Keywords:** spinal cord injury, glial scar, astrocyte, scar-forming astrocyte, regeneration

The article (Silver, 2016) was not cited in the published version of our article. The citation has now been inserted in section The Dual Role of the Glial Scar in SCI Recovery, subsection Scar-Forming Astrocytes Exhibit Environment-Dependent Plasticity and Could Serve as Bridges for Axonal Regrowth Under Certain Conditions, paragraph two, and should read:

“The bridge formed by astrocytes is more like a short “drawbridge” (Silver, 2016).”

The authors apologize for this error and state that this does not change the scientific conclusions of the article in any way. The original article has been updated.
